# Diversity and distribution of freshwater copepods (Calanoida, Cyclopoida, Harpacticoida) in Thailand

**DOI:** 10.3897/BDJ.14.e212289

**Published:** 2026-10-29

**Authors:** Thanida Saetang, Supiyanit Maiphae

**Affiliations:** 1 Biodiversity Center Kasetsart University (BDCKU), Kasetsart University, Bangkok, Thailand Biodiversity Center Kasetsart University (BDCKU), Kasetsart University Bangkok Thailand; 2 Department of Fishery Biology, Faculty of Fisheries, Kasetsart University, Bangkok, Thailand Department of Fishery Biology, Faculty of Fisheries, Kasetsart University Bangkok Thailand; 3 Animal Systematics and Ecology Speciality Research Unit (ASESRU), Department of Zoology, Faculty of Science, Kasetsart University, Bangkok, Thailand Animal Systematics and Ecology Speciality Research Unit (ASESRU), Department of Zoology, Faculty of Science, Kasetsart University Bangkok Thailand

**Keywords:** biogeography, habitat specificity, species accumulation, species richness

## Abstract

Freshwater copepods are an important component of aquatic biodiversity in Thailand, but information on their diversity and distribution is still scattered across taxonomic and ecological studies. We compiled and reviewed published records of freshwater copepods from Thailand, revised nomenclature and taxonomic status and analysed species richness, habitat associations, geographical and zoogeographical distributions and inventory completeness. The dataset included 21,505 occurrence records from 115 publications, describing 187 species in 60 genera. Of these, 67 species (35.8%) were originally described from Thailand and 58 species (31.0%) have so far been recorded only from Thailand according to the available literature. The most species-rich families were Cyclopidae (82 species, 43.9%), Diaptomidae (47 species, 25.1%) and Canthocamptidae (28 species, 15.0%). Species richness varied markedly amongst habitats and geographical regions. Ponds supported the highest richness (75 species), closely followed by cave pools (70 species) and north-eastern Thailand had the highest regional richness (103 species). Cave pools also contained the most habitat-restricted species (36 species). Only 11 species (5.9%) were recorded from all six geographical regions, whereas 102 species were restricted to a single geographical region and 94 species (50.3%) were restricted to the Oriental Region. The species accumulation curve did not reach an asymptote and non-parametric estimators predicted 220–346 species, with estimated sampling completeness ranging from 54.1% to 85.0%. These findings indicate that Thailand's freshwater copepod fauna remains incompletely documented despite extensive research. Continued surveys, particularly in underexplored geographical regions and in habitats that yielded few species, such as groundwater and cave streams, are needed to improve estimates of copepod diversity and distribution in Thailand.

## Introduction

Thailand is recognised as one of the biodiversity hotspots in Southeast Asia, supporting highly diverse freshwater zooplankton communities due to the wide variety of aquatic habitats distributed throughout the country (Sanoamuang 1999, Maiphae et al. 2023, Saetang and Maiphae 2023). Amongst freshwater zooplankton, copepods are a species-rich and ecologically important group in inland aquatic ecosystems. They occupy a wide range of habitats, including lakes, ponds, rivers, swamps, peat swamps, rice fields, temporary waters, cave pools and groundwater systems, where they play important roles in aquatic food webs and nutrient cycling (Boxshall and Jaume 2000, Boxshall and Defaye 2008). Many Thai freshwater copepods also exhibit high habitat specificity and restricted geographical distributions, reflecting their close associations with particular environmental conditions (Sanoamuang 1999, Watiroyram et al. 2015a).

Research on Thai freshwater copepods began several decades ago and increased substantially from the 1990s onwards, particularly in taxonomy, biodiversity and biogeography (Boonsom 1984, Sanoamuang 1999). During the last decade, research has expanded considerably into integrative taxonomy and biodiversity surveys of previously understudied habitats, including temporary ponds, rice fields, caves and swamps (Watiroyram et al. 2015b, Boonyanusith et al. 2018a, Boonyanusith et al. 2018b, Saetang et al. 2021, Saetang et al. 2022, Saetang and Maiphae 2023). Despite these efforts, much of the available information remains based on species descriptions and localised records, while broader syntheses of species richness, habitat associations and geographical distribution throughout Thailand remain limited. Consequently, large-scale patterns of copepod diversity and distribution in tropical freshwater ecosystems are still poorly understood.

So far, 170 freshwater copepod species have been reported from Thailand, with Cyclopidae representing the most diverse family (Saetang and Maiphae 2023). Recent investigations in diverse aquatic habitats have continued to reveal new species and new distributional records. For example, four new species of *Tropodiaptomus* – *T. megahyaline*, *T. longiprocessus*, *T. pedecrassum*, and *T. sirindhornae* — have recently been described from freshwater habitats in Thailand (Saetang et al. 2021, Saetang and Maiphae 2023, Saetang and Maiphae 2025), a new *Thermocyclops* species was discovered in an agricultural rice field (Saetang et al. 2024) and highly specialised copepod faunas have been documented from subterranean and cave systems (Watiroyram et al. 2015a, Watiroyram et al. 2022, Watiroyram and Koompoot 2023, Boonyanusith et al. 2024). Furthermore, integrative taxonomic approaches combining morphological and molecular data have revealed substantial genetic diversity and cryptic species complexes amongst Thai copepods (Saetang et al. 2022). These continued discoveries suggest that the currently known copepod fauna of Thailand remains incompletely documented.

Despite decades of accumulated research, a comprehensive synthesis of Thai freshwater copepods is still lacking. Much of the available information remains fragmented across localised publications and the available records differ amongst geographical regions and habitat types. In addition, the nomenclature and the taxonomic status of some species reported in earlier studies have changed following recent taxonomic revisions. An updated synthesis is, therefore, needed to consolidate existing knowledge, evaluate the completeness of the current species inventory and identify geographical and habitat-level patterns in copepod diversity.

To address these knowledge gaps, this study provides an updated checklist and synthesis of freshwater copepods (Calanoida, Cyclopoida and Harpacticoida) in Thailand, based on an extensive literature review and recent taxonomic advances. We further analyse species richness, habitat associations, geographical distribution and zoogeographical patterns of the recorded fauna. This updated synthesis contributes to a more comprehensive understanding of freshwater copepod diversity in tropical Asia and identifies priority areas and habitats for future taxonomic, ecological and biogeographical research.

## Material and methods

A checklist of freshwater copepod species in Thailand was compiled from 89 published research papers, 18 theses and eight reports available up to 2026 (Table 1). Updated nomenclature and current taxonomic status were verified using the World of Copepods Database through the World Register of Marine Species (WoRMS Editorial Board 2026), the Copepod Web Portal (Pesce 2024) and relevant recent taxonomic revisions and literature. Previously used species names and synonyms reported in earlier publications were also reviewed and updated when necessary.

Data on habitat occurrence, geographical distribution within Thailand and biogeographical distribution were extracted from literature. For each species, reported distribution records were reviewed and assigned to the major biogeographical regions following Balian et al. (2008), including the Palaearctic, Afrotropical, Oriental, Nearctic, Neotropical, Australasian, Pacific Oceanic Islands and Antarctic regions. Species occurring in more than one region were assigned to all relevant regions. Records that could not be assigned to a specific biogeographical region because of insufficient information were treated as unresolved. Species with a broad worldwide distribution were classified as cosmopolitan when supported by the available literature.

For data analyses, Thailand was divided into six geographical regions: north, northeast, west, east, central and south, following the geographical classification of Thailand (Office of the Royal Society 2020). Habitat types were compiled from published records of copepod species in Thailand and classified into 19 categories: surface habitats comprised canal, check dam, dam, floodplain wetland, lake, mine, pond, pool, peat swamp, reservoir, rice field, river, stream and swamp, whereas subterranean water habitats comprised large persistent cave waterbody, cave pool, cave stream, cave habitat of undetermined hydrological type and groundwater/well. Some habitat categories may partially overlap because the original habitat terms were retained.

Species richness estimators, including Chao2, Jackknife1, Jackknife2 and Bootstrap, were calculated using species accumulation curves to evaluate the completeness of copepod diversity records in Thailand. The Jaccard similarity index was used to assess similarities in copepod species composition amongst habitat types. All analyses were performed using R version 4.6.1 (R Core Team 2026).

## Results

The results are presented in two main aspects: (1) species richness, including variation amongst taxonomic groups and habitat types and (2) geographical distribution within Thailand and zoogeographical regions.

### Species richness

The dataset comprised 21,505 occurrence records of freshwater copepods compiled from 115 publications (Suppl. material 1). Overall, three orders, Calanoida (5 families), Cyclopoida (3 families) and Harpacticoida (8 families), were recorded, representing 60 genera and 187 species in Thailand. Of these, 67 species (35.8%) were originally described from Thailand (23 calanoids, 19 cyclopoids and 25 harpacticoids), whereas 58 species (31.0%) are currently known only from Thailand, based on published records (Table 2).

Species richness was unevenly distributed amongst families. Cyclopidae was the most species-rich family (82 species), followed by Diaptomidae (47 species) and Canthocamptidae (28 species). The remaining families were represented by comparatively few species, including Laophontidae (6 species), Ameiridae (5 species), Phyllognathopodidae (4 species), Parastenocarididae (3 species), Acartiidae, Halicyclopidae and Ectinosomatidae (2 species each), whereas Centropagidae, Pseudocyclopidae, Pseudodiaptomidae, Cyclopettidae, Miraciidae and Tegastidae were each represented by a single species.

**Figure 1.**
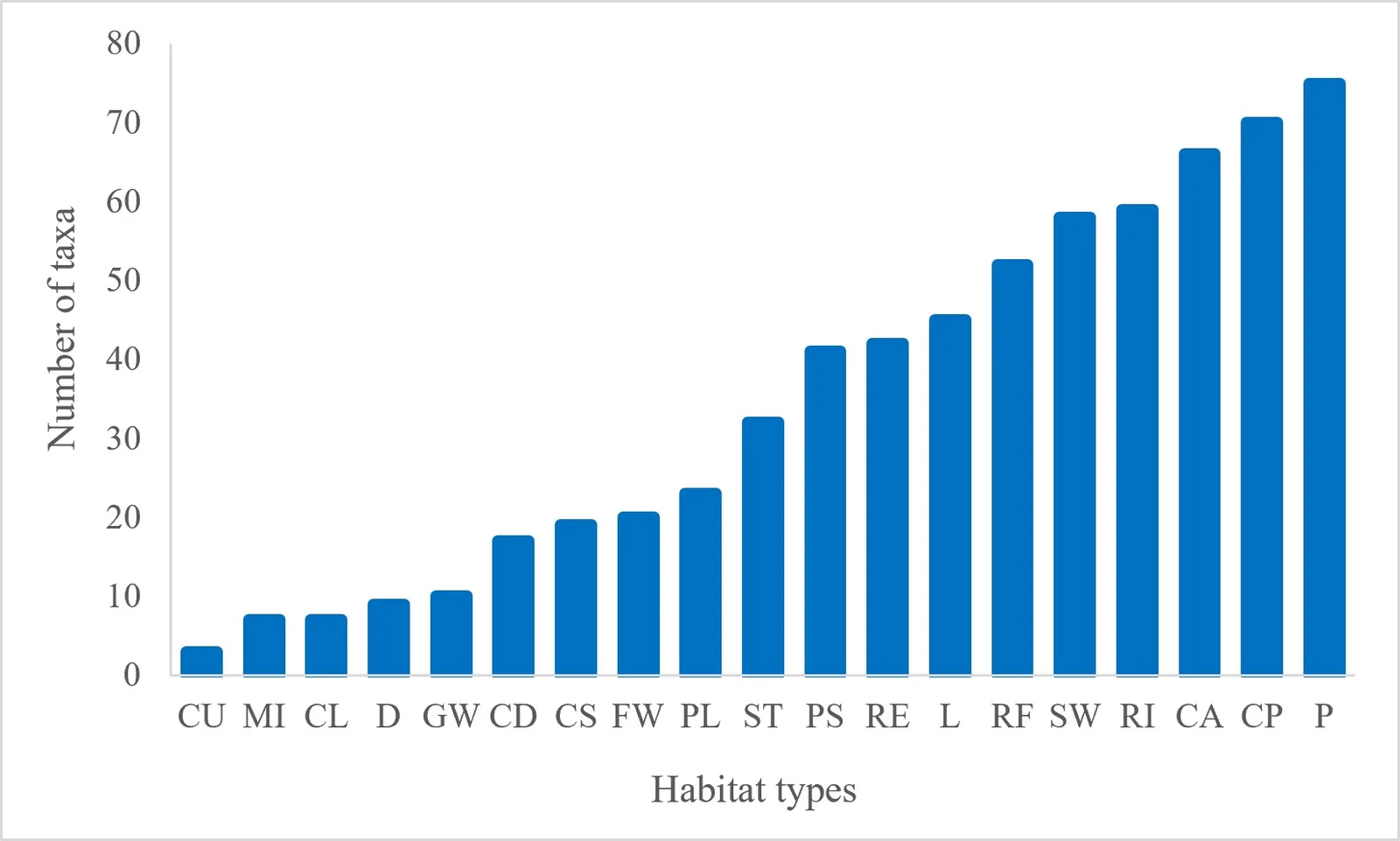
Species richness of freshwater copepods across different habitat types in Thailand. Abbreviations are as shown in Table 2.

The distribution of copepod species richness varied markedly amongst habitat types. Species richness ranged from three species in cave habitats of undetermined hydrological type to 75 species in ponds. Only six habitat types supported more than 50 recorded species, whereas the majority contained fewer than 50 species. Ponds harboured the highest species richness (75 species), closely followed by cave pools (70 species), canals (66 species), rivers (59 species), swamps (58 species) and rice fields (52 species) also supported relatively high species richness. Intermediate richness was recorded in lakes (45 species), reservoirs (42 species), peat swamps (41 species), streams (32 species), pools (23 species), floodplain wetlands (20 species), cave streams (19 species) and check dams (17 species). The remaining habitat types supported 10 or fewer species, including groundwater/wells (10 species), dams (9 species), mines and large persistent cave waterbodies (7 species each) and cave habitats of undetermined hydrological type (3 species), which exhibited the lowest recorded species richness (Fig. 1). However, because the data were compiled from studies with different sampling designs and efforts, the recorded species richness should be interpreted as cumulative documented richness and may partly reflect differences in sampling intensity and the number of available studies amongst habitat types.

**Figure 2.**
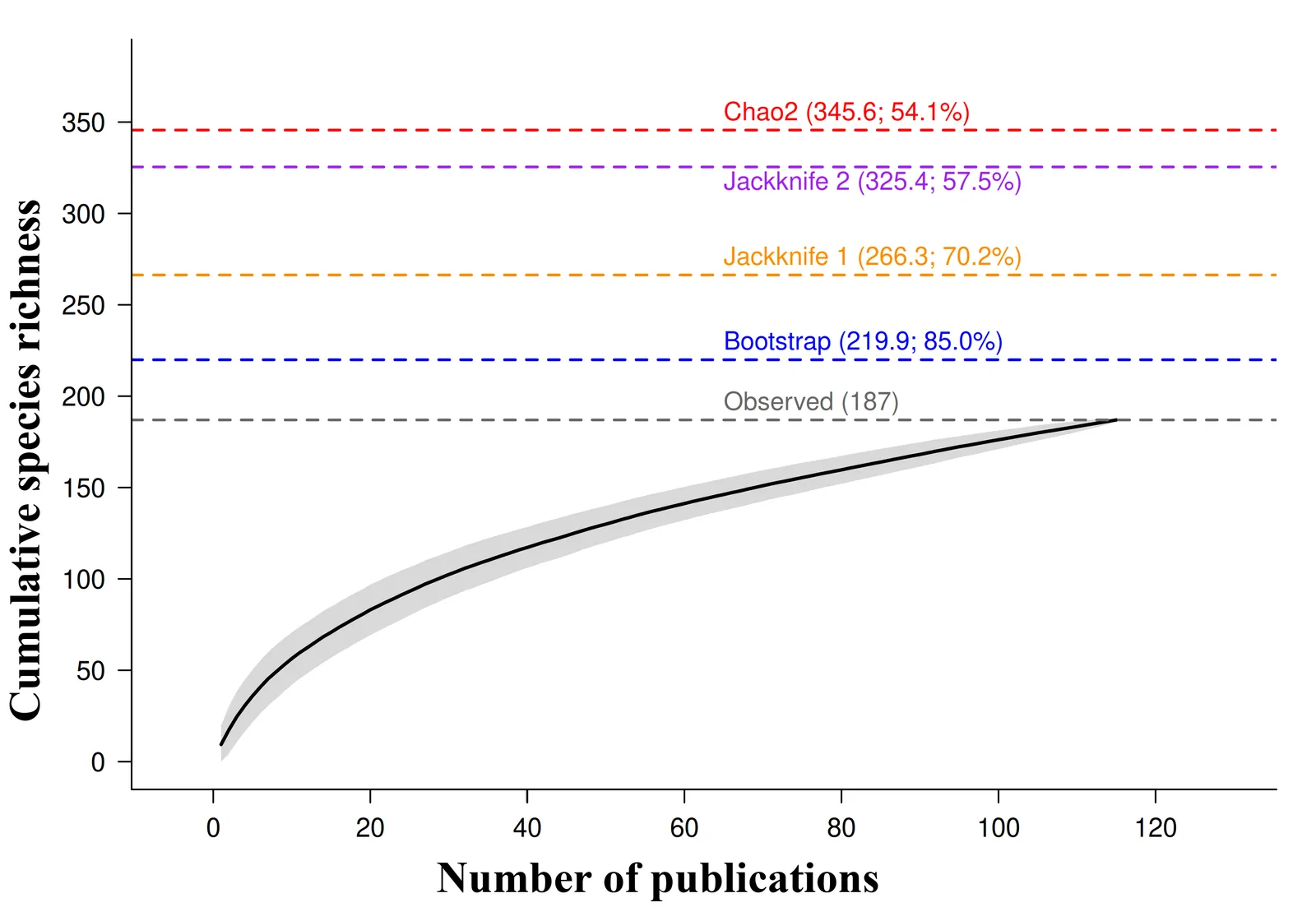
Species accumulation curve of freshwater copepods in Thailand, based on the number of publications.

The species accumulation curve increased rapidly during the early stages of published records, but gradually levelled off as the number of publications increased. However, the curve did not reach an asymptote. The observed richness was 187 species, whereas all non-parametric richness estimators predicted higher values, ranging from 220 species (Bootstrap) to 346 species (Chao2). Estimated sampling completeness ranged from 54.1% (Chao2) to 85.0% (Bootstrap), indicating that the current checklist represents approximately 54–85% of the predicted copepod diversity in Thailand (Fig. 2).

### Distribution

**Figure 3.**
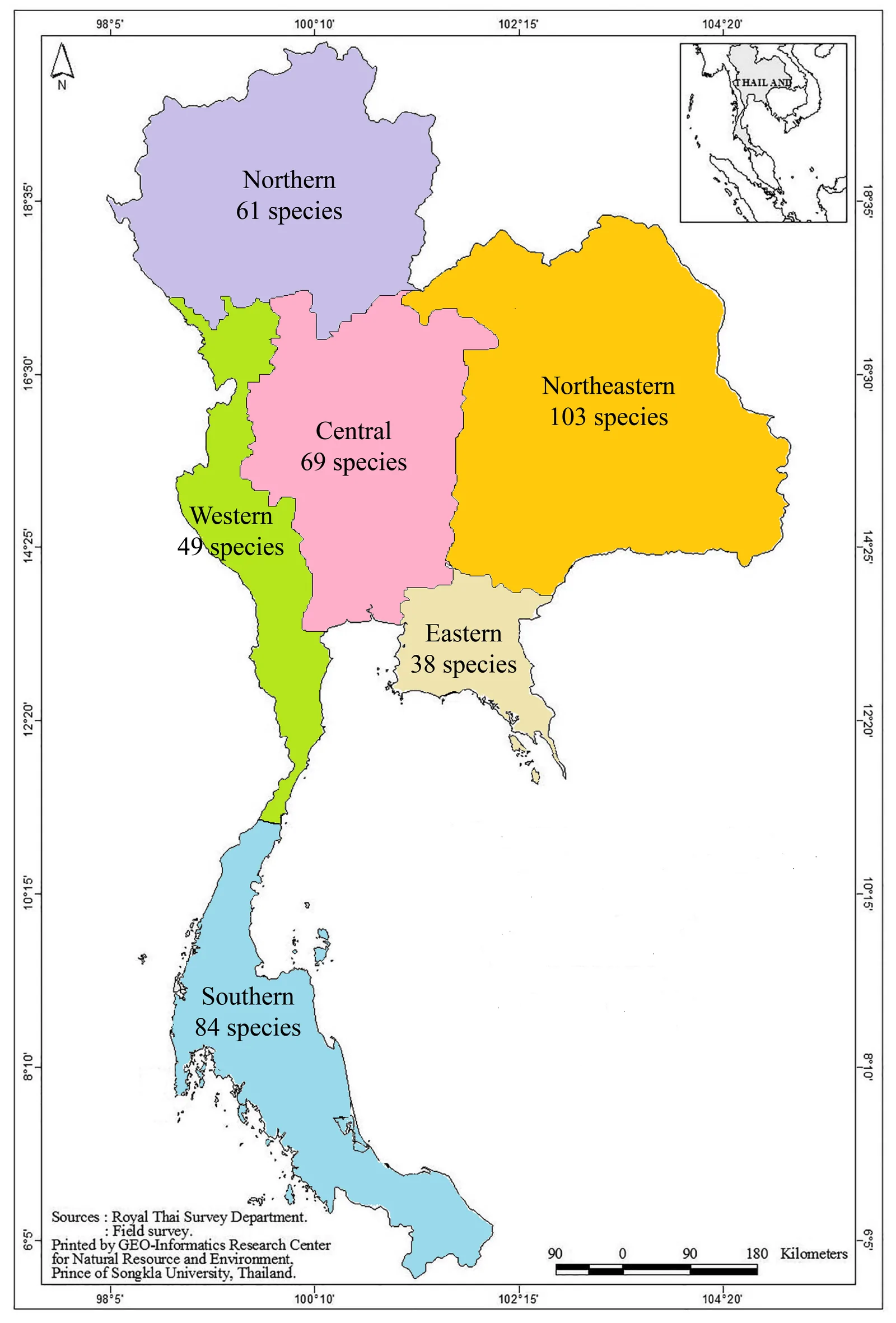
Species richness of freshwater copepods across the six geographical regions of Thailand.

Copepod species richness varied considerably amongst the six geographical regions of Thailand. Species richness ranged from 38 species in eastern Thailand to 103 species in north-eastern Thailand. North-eastern Thailand harboured the highest species richness (103 species), followed by southern Thailand (84 species), central Thailand (69 species), northern Thailand (61 species) and western Thailand (49 species), whereas eastern Thailand contained the fewest recorded species (38 species) (Fig. 3).

Most copepod species exhibited restricted geographical distributions. Only 11 species (5.9%) were recorded from all six geographical regions of Thailand: *Eucyclops serrulatus*, *Mesocyclops affinis*, *M. aspericornis*, *M. ogunnus*, *M. thermocyclopoides*, *Microcyclops varicans*, *Mongolodiaptomus botulifer*, *M. malaindosinensis*, *Phyllodiaptomus praedictus*, *Thermocyclops crassus* and *Tropodiaptomus vicinus*. In contrast, 102 species (54.5%) were restricted to a single geographical region. North-eastern Thailand contained the highest number of regionally restricted species (38 species, 20.3%), followed by southern Thailand (31 species, 16.6%). Central, northern, western and eastern Thailand harboured 13 (7.0%), 10 (5.3%), 6 (3.2%), and 4 (2.1%) regionally restricted species, respectively (Fig. 3, Table 2).

**Figure 4.**
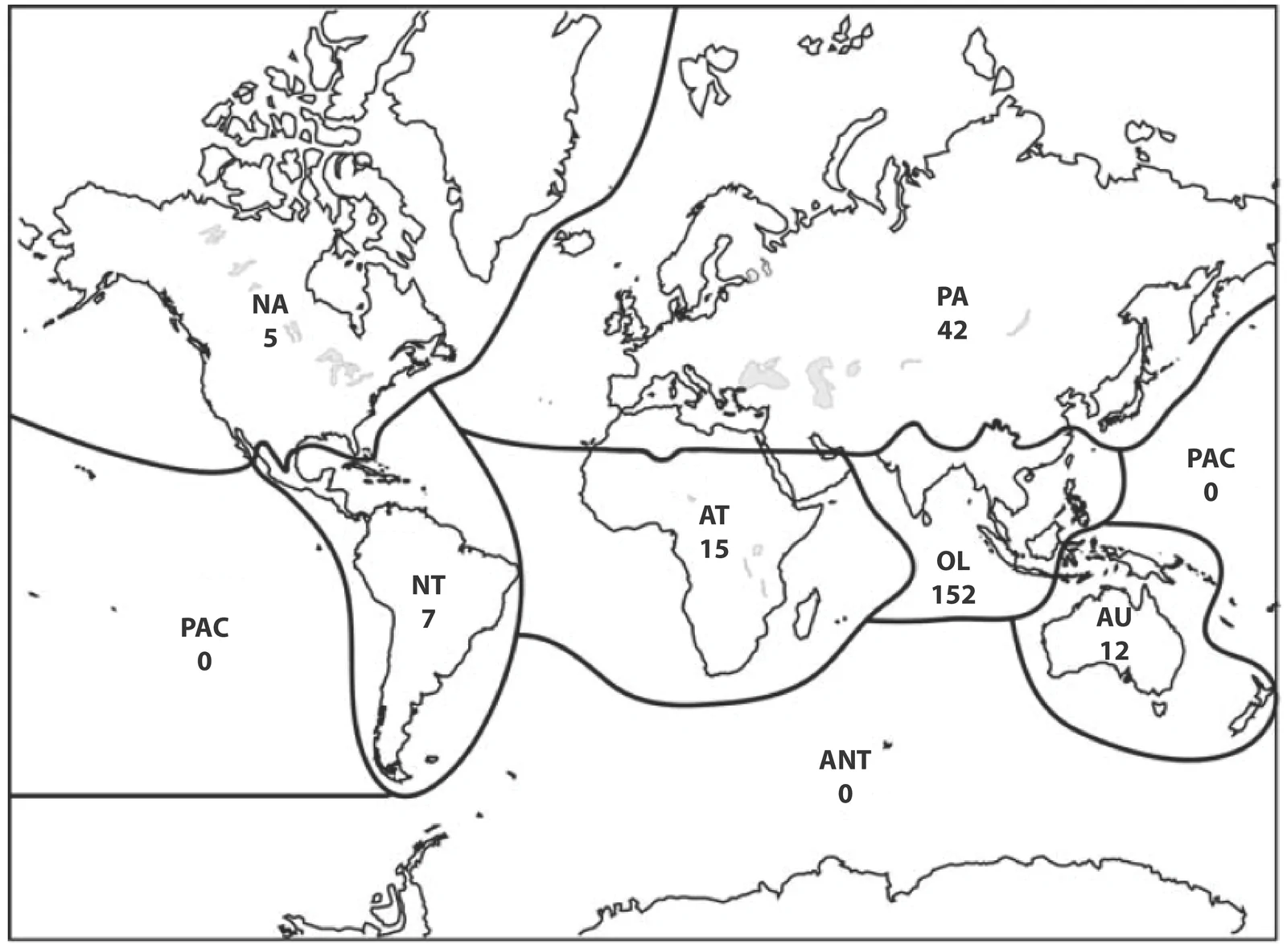
Zoogeographical distribution of freshwater copepod species recorded in Thailand. Numbers indicate the number of species recorded in Thailand that occur in each zoogeographical region. Cosmopolitan species and species with unresolved zoogeographical distributions are excluded. Abbreviations are as shown in Table 2.

Beyond their distribution within Thailand, the recorded species showed distinct zoogeographic patterns (Fig. 4). Of the 187 species, 58 (31.0% of the total fauna) were known only from Thailand, while 94 species (50.3%) were restricted to the Oriental Region. The remaining 58 species occurred in the Oriental Region and at least one other zoogeographical region. Of these, 39 species (20.9%) were distributed in two zoogeographic regions, 15 species (8.0%) in three regions and four species (2.1%) in four regions. Eighteen taxa (9.6%) were classified as cosmopolitan. The remaining 17 species (9.1%) could not be assigned to a zoogeographic region because their taxonomic status remains unresolved. The specific regional combinations for each species are provided in Table 2.

Habitat-restricted species were unevenly distributed amongst habitat types. Overall, 89 species (47.6% of the total fauna) were recorded from only one habitat type. Cave pools contained by far the highest number of habitat-restricted species (36 species; 19.2% of the total fauna and 51.4% of all species recorded from cave pools), followed by canals and ponds (7 species each; 3.7%), lakes and pools (6 species each; 3.2%), cave streams and groundwater/wells (5 species each; 2.7%) and swamps (4 species; 2.1%). Peat swamps and streams each contained three species (1.6%), while reservoirs and cave habitats of undetermined hydrological type each contained two species (1.1%). Large persistent cave waterbodies, rivers and rice fields each harboured a single species (0.5%). No species were restricted exclusively to mines, dams, check dams or floodplain wetlands.

Pairwise comparisons of species composition revealed the greatest similarity between rice fields and rivers (65.7%), followed by canals and rice fields (61.6%), ponds and rivers (61.4%) and canals and ponds (58.4%) (Suppl. material 2).

## Discussion

### Species richness

The species accumulation curve did not show a clear plateau, as cumulative species richness continued to increase towards the end of the curve and all non-parametric estimators predicted higher species richness than the number of currently recorded species. Species accumulation curves and non-parametric richness estimators are widely used to assess inventory completeness and estimate undetected species in biodiversity datasets (Colwell and Coddington 1994, Chao et al. 2014). Depending on the estimator used, the current checklist represents approximately 54–85% of the predicted copepod diversity, suggesting that additional species may remain to be documented through future surveys and taxonomic studies. However, because these estimates are based on secondary data compiled from studies with different sampling designs and efforts, they should be interpreted with caution. A similar pattern has also been observed in other freshwater zooplankton groups in Thailand, including cladocerans and rotifers, for which updated national checklists indicate that their diversity is still incompletely documented (Choedchim and Maiphae 2023, Jaturapruek and Maiphae 2025). This suggests that gaps in biodiversity documentation are not unique to copepods and that further surveys and taxonomic studies are still needed, particularly in habitat types from which few species were recorded in the present study, such as cave habitats of undetermined hydrological type, large, persistent cave waterbodies, mines, dams and groundwater. Subterranean habitats other than cave pools, including cave streams, also warrant attention, as they yielded comparatively few species relative to cave pools supplied by dripping, seeping or percolating water and to the most species-rich surface habitats.

The apparent incompleteness of the current inventory may partly reflect the diversity of freshwater habitat types across Thailand, which encompasses a wide range of ecological conditions and can support distinct copepod assemblages (Tews et al. 2004, Stein et al. 2014). In the present study, species richness varied markedly amongst habitat types, with ponds, cave pools and canals supporting the highest numbers of species, whereas most other subterranean habitats were comparatively species-poor. Cave pools were the only subterranean habitat with richness comparable to that of the most species-rich surface habitats and they supported 36 habitat-restricted species, representing 51.4% of the copepod fauna recorded from this habitat. Such habitat-specific distributions can contribute to a high proportion of rare or unique records and may, therefore, reduce the apparent completeness of the overall inventory. Recent investigations of subterranean habitats, particularly cave pools, have contributed to the discovery of numerous previously undocumented copepod species in Thailand (e.g. Karanovic et al. (2017), Watiroyram and Sanoamuang (2017), Boonyanusith et al. (2018b), Watiroyram (2018a), Boonyanusith et al. (2020), Koompoot and Sanoamuang (2021), Watiroyram (2021b), Athibai et al. (2022), Watiroyram et al. (2022), Boonyanusith et al. (2024)). A similar relationship between habitat diversity and species richness has been reported for freshwater cladocerans in Thailand, where different habitat types supported distinct assemblages and less-explored habitats were identified as important areas for future surveys (Choedchim and Maiphae 2023). The comparison with cladocerans, therefore, highlights the importance of sampling a broad range of habitat types for obtaining a more complete picture of freshwater zooplankton diversity in Thailand. For copepods, this pattern is particularly evident in subterranean habitats, where species distributions can be highly restricted, as documented both globally and in cave systems in Thailand (Galassi et al. 2009, Watiroyram et al. 2021). The low richness recorded from some habitat types, particularly groundwater and less accessible cave habitats, may reflect limited sampling rather than genuinely low diversity.

In addition to habitat diversity, sampling effort, sampling methods and differences in taxonomic effort amongst copepod groups may have contributed to the observed pattern. Compared with calanoid and cyclopoid copepods, intensive taxonomic studies of harpacticoid copepods, especially those inhabiting subterranean environments, have expanded only in recent years and continue to reveal previously undocumented species. Continued exploration of specialised habitats, together with integrative taxonomic approaches combining morphological and molecular evidence (Dayrat 2005, Padial et al. 2010, Saetang et al. 2022), is likely to further increase the known diversity of freshwater copepods in Thailand. Moreover, because the number of species recorded from each habitat type is influenced by the number of sites and samples collected, the differences in richness amongst habitat types observed here should be interpreted with caution.

Differences in species richness amongst copepod families may be associated with both their ecological characteristics and differences in taxonomic and sampling effort. The higher number of species recorded in some families, such as Diaptomidae and Canthocamptidae, may be related to their wider habitat distribution and tolerance of different environmental conditions, as well as the greater attention given to these groups in previous taxonomic studies. Therefore, differences in species richness at the family level may not fully represent the actual diversity of each copepod family in Thailand. Further surveys, particularly in poorly studied regions and habitats, together with additional taxonomic studies, are needed to improve our knowledge of freshwater copepod diversity and species distributions in Thailand.

### Distribution

Marked geographical variation in species richness was evident amongst the six regions of Thailand. North-eastern Thailand had the highest overall species richness and the greatest number of regionally restricted species, whereas eastern Thailand had the fewest recorded species. This pattern may be related to differences in habitat diversity and historical sampling effort. North-eastern Thailand contains a wide variety of freshwater habitats, including ponds, reservoirs, wetlands, rice fields and temporary waterbodies, which provide different environmental conditions suitable for freshwater copepods (Sanoamuang 1999, Sanoamuang and Dabseepai 2021). In addition, freshwater habitats in this region have been studied over several decades, resulting in a larger accumulation of species records. Long-term sampling may have increased the likelihood of detecting species with restricted distributions and those occurring in less frequently studied habitats. Thus, the high species richness recorded in north-eastern Thailand may reflect both its diverse freshwater habitats and its longer history of taxonomic and biodiversity surveys. However, the relative contributions of habitat diversity and sampling effort cannot be clearly distinguished from the available data. A similar geographical pattern has been reported for freshwater rotifers, with north-eastern Thailand supporting the highest species richness, followed by southern Thailand. This pattern was also associated with differences in habitat diversity and sampling intensity amongst regions (Jaturapruek and Maiphae 2025).

In addition, most copepod species exhibited restricted geographical distributions within Thailand. Only 11 species (5.9%) were recorded from all six geographical regions, whereas 102 species (54.5%) were confined to a single region. Such spatial turnover may reflect regional environmental heterogeneity, historical biogeography and dispersal limitation, although unequal sampling intensity may also partly explain the observed patterns (Leibold et al. 2010, Heino 2011).

The zoogeographical composition of the Thai freshwater copepod fauna further supports this interpretation. A total of 94 species (50.3% of the recorded species) were restricted to the Oriental Region, whereas 58 species (31.0%) were distributed in multiple zoogeographical regions and only 18 species (9.6%) were classified as cosmopolitan. The high proportion of species restricted to the Oriental Region is consistent with Thailand's geographical position in tropical Southeast Asia. This pattern may also reflect the geological and climatic history of the region, which has shaped freshwater biogeographic patterns (Wallace 1876, Morrone 2015).

Compared with freshwater rotifers, however, Thai freshwater copepods exhibit a much stronger Oriental affinity. Whereas rotifer assemblages are dominated by cosmopolitan species with relatively few Oriental endemics (Jaturapruek and Maiphae 2025), nearly half of the copepod species are restricted to the Oriental Region. This contrast probably reflects fundamental differences in dispersal ability and life-history traits between the two groups. Rotifers can be readily dispersed through resistant resting stages, facilitating long-distance passive dispersal, whereas freshwater copepods generally have more limited dispersal opportunities across major geographical barriers. Their distributions are, therefore, more strongly constrained by drainage connectivity, habitat specialisation and historical biogeographical processes (Boxshall and Jaume 2000, Boxshall and Defaye 2008). Consequently, many copepod species remain confined to particular biogeographical regions or freshwater systems.

The observed distribution patterns, characterised by numerous regionally restricted species and a strong Oriental affinity, indicate that freshwater copepod diversity in Thailand has been shaped by processes operating at multiple spatial scales. At the national scale, differences in habitat availability and environmental conditions may contribute to regional variation in species composition, whereas at the continental scale, historical biogeographical processes have likely influenced the overall composition of the fauna. Future surveys should, therefore, prioritise both geographically under-sampled regions, particularly eastern and western Thailand and ecologically specialised habitats such as springs, groundwater systems, peat swamps and cave pools, where additional distribution records and previously undocumented species are likely to be discovered. Such targeted sampling, together with the integration of morphological and molecular approaches (Dayrat 2005, Padial et al. 2010, Saetang et al. 2022), will improve estimates of copepod diversity, refine our understanding of species distributions and provide a stronger basis for future biogeographical and conservation studies of Thailand's freshwater ecosystems.

## Conclusions

Overall, the present checklist demonstrates that Thailand supports a diverse freshwater copepod fauna, characterised by substantial taxonomic, ecological, and biogeographical variation. The combination of high habitat diversity, marked regional variation in species richness and distribution, a predominance of Oriental elements and continuing taxonomic discoveries, particularly from subterranean habitats, indicates that the currently documented fauna remains an underestimate of the actual copepod diversity of the country. The incomplete species accumulation curve and richness estimates further suggest that additional species remain to be documented. Continued biodiversity surveys, especially in under-represented regions and habitats, together with integrative taxonomic research, will be essential for improving our understanding of the diversity, distribution and biogeographical history of freshwater copepods in Thailand.

## Supplementary Material

B1B6F2D6-585D-51E2-87EF-5FAF38B4E43F 10.3897/BDJ.14.e212289.suppl1 Supplementary material 1 Presence-absence matrix of copepod species Data type Occurrence data (presence–absence) Brief description Presence-absence records of copepod species compiled from literature sources, where 1 indicates presence and 0 indicates absence, used for species richness estimation in Thailand. File: oo_1804480.xlsx https://binary.pensoft.net/file/1804480 Thanida Saetang and Supiyanit Maiphae

16CC0D2C-0CF8-5AA0-B661-8711255A4A0C 10.3897/BDJ.14.e212289.suppl2 Supplementary material 2 Pairwise Jaccard similarity amongst habitat types Data type Similarity data Brief description Pairwise Jaccard similarity values (%) amongst habitat types, calculated from copepod species presence–absence data. File: oo_1796529.xlsx https://binary.pensoft.net/file/1796529 Thanida Saetang and Supiyanit Maiphae

## Figures and Tables

**Table 1.** Reference codes and corresponding references cited in Table 2.

**Reference code**	**Reference**	**Reference code**	**Reference**
1	Daday (1906)	59	Saetang and Maiphae (2015a)
2	Bricker et al. (1978)	60	Saetang and Maiphae (2015b)
3	Lai and Fernando (1981)	61	Watiroyram et al. (2015a)
4	Boonsom (1984)	62	Watiroyram et al. (2015b)
5	Pesce and Apostolov (1985)	63	Saetang (2016)
6	Dumont and Ranga Reddy (1994)	64	Watiroyram and Brancelj (2016)
7	Sanoamuang (1994)	65	Karanovic et al. (2017)
8	Dumont et al. (1996)	66	Maiphae (2017)
9	Maiphae (1997)	67	Watiroyram and Sanoamuang (2017)
10	Pholpunthin (1997)	68	Watiroyram et al. (2017)
11	Ranga Reddy and Dumont (1998)	69	Boonyanusith et al. (2018a)
12	Ranga Reddy et al. (1998)	70	Boonyanusith et al. (2018b)
13	Ranga Reddy and Dumont (1999)	71	Sanoamuang and Watiroyram (2018)
14	Sanoamuang (1999)	72	Watiroyram (2018a)
15	Ranga Reddy et al. (2000)	73	Watiroyram (2018b)
16	Sanoamuang (2001a)	74	Watiroyram (2018c)
17	Sanoamuang (2001b)	75	Koompoot (2019)
18	Sanoamuang (2001c)	76	Boonyanusith et al. (2020)
19	Sanoamuang and Yindee (2001)	77	Janpong and Sanoamuang (2020)
20	Savatenalinton (2001)	78	Sanoamuang and Watiroyram (2020)
21	Athibai (2002)	79	Boonyanusith and Athibai (2021)
22	Sanoamuang and Athibai (2002)	80	Koompoot and Sanoamuang (2021)
23	Yindee (2002)	81	Plangklang and Athibai (2021)
24	Lekchan (2003)	82	Saetang (2021)
25	Tungpunyaporn (2003)	83	Saetang et al. (2021)
26	Wongrat and Pipatcharoenchai (2003)	84	Sanoamuang and Dabseepai (2021)
27	Faitakum (2004)	85	Sanoamuang and Watiroyram (2021a)
28	Sanoamuang (2004)	86	Sanoamuang and Watiroyram (2021b)
29	Sanoamuang and Sivongxay (2004)	87	Watiroyram (2021a)
30	Wansuang (2004)	88	Watiroyram (2021b)
31	Sanoamuang and Faitakum (2005)	89	Watiroyram et al. (2021)
32	Alekseev and Sanoamuang (2006)	90	Athibai et al. (2022)
33	Boonsit (2006)	91	Boonyanusith et al. (2022a)
34	Jithlang and Wongrat (2006)	92	Boonyanusith et al. (2022b)
35	Proongkiat (2006)	93	Saetang et al. (2022)
36	Sanoamuang and Teeramaethee (2006)	94	Watiroyram et al. (2022)
37	Teeramaethee (2006)	95	Boonyanusith and Wongkamhaeng (2023)
38	Wansuang and Sanoamuang (2006)	96	Maiphae et al. (2023)
39	Apostolov (2007)	97	Saetang and Maiphae (2023)
40	Inpang (2008)	98	Sanoamuang and Watiroyram (2023)
41	Proongkiat and Sanoamuang (2008)	99	Soe and Sanoamuang (2023)
42	Chittapun et al. (2009)	100	Watiroyram and Koompoot (2023)
43	Chullasorn et al. (2009)	101	Boonyanusith et al. (2024)
44	Brancelj et al. (2010)	102	Brancelj et al. (2024)
45	Cottarelli et al. (2010)	103	Maiphae and Saetang (2024)
46	Koompoot (2010)	104	Saetang et al. (2024)
47	Maiphae et al. (2010)	105	Sanoamuang and Koompoot (2024)
48	Suankamjad (2011)	106	Watiroyram and Koompoot (2024)
49	Koompoot and Sanoamuang (2012)	107	Boonyanusith et al. (2025)
50	Pholpunthin et al. (2012)	108	Koompoot and Sanoamuang (2025)
51	Saetang (2012)	109	Koompoot and Watiroyram (2025)
52	Watiroyram (2012)	110	Saetang and Maiphae (2025)
53	Watiroyram et al. (2012)	111	Watiroyram et al. (2025)
54	Boonyanusith (2013)	112	Boonmak et al. (2026)
55	Boonyanusith et al. (2013)	113	Koompoot and Phlai-Ngam (2026)
56	Boonyanusith and Athibai (2014)	114	Saetang and Maiphae (2026)
57	Bruno and Cottarelli (2015)	115	Sanoamuang et al. (2026)
58	Boonyanusith and Wongkamhaeng (2015)		

**Table 2.** Checklist of copepods recorded in Thailand, with habitat types, based on published records from Thailand, geographical distribution of Thailand and zoogeographical regions. Notes: * = endemic to Thailand. - = information unavailable or not reliably determined from the available literature. **Surface habitat types**: CA = canal, CD = check dam, D = dam, FW = floodplain wetland, L = lake, MI = mine, P = pond, PL = pool, PS = peat swamp, RE = reservoir, RF = rice field, RI = river, ST = stream, SW = swamp. Subterranean water habitats: CL = large, persistent cave waterbody with standing or very slow-flowing water; CP = cave pool supplied by dripping, seeping or percolating water; CS = cave stream, including karst springs discharging from saturated karstic aquifers; CU = cave habitat of undetermined hydrological type; GW = groundwater/well. **Geographical regions of Thailand**: C = central, E = east, N = north, NE = northeast, S = south, W = west. **Zoogeographical regions**: ANT = Antarctic, AT = Afrotropical, AU = Australasian, NA = Nearctic, NT = Neotropical, OL = Oriental, PA = Palaearctic, PAC = Pacific Oceanic Islands. Reference codes correspond to those listed in Table 1.

	**Taxa**	**Habitat types**	**Geographical regions of Thailand**	**Zoogeographical regions**	**References**
	**Order Calanoida Sars G.O., 1903**				
	**Family Acartiidae Sars G.O., 1903**				
1	*Acartiella sinensis* Shen & Lee, 1963	L	S	NA, OL, PA	10, 40, 59, 63
2	Acartia cf. southwelli	L	S	-	40
	**Family Centropagidae Giesbrecht, 1893**				
3	*Sinocalanus tenellus* (Kikuchi, 1928)	CA	C	OL, PA	3
	**Family Diaptomidae Baird, 1850**				
4	*Allodiaptomus raoi* Kiefer, 1936	CD, L, P, RE, RI, RF, SW	NE	OL, PA	2, 4, 12, 14, 33, 37, 84, 109, 114
5	*Arctodiaptomus* sp.	RI	NE	-	84
6	*Dentodiaptomus javanus* (Grochmalicki, 1915) Remark: Previously recorded as *Diaptomus javanus* (Lai and Fernando, 1981).	CA, FW, P, RE, RF, RI, ST, SW	E, N, NE, W	OL	3, 14, 16, 18, 19, 21, 22, 23, 24, 25, 27, 28, 29, 30, 31, 33, 36, 38, 46, 49, 71, 82, 84, 109, 111, 114, 115
7	*D. orientalis* Sanoamuang & Watiroyram, 2021	CA, P	NE	OL	84, 86
8	*Eodiaptomus draconisignivomi* Brehm, 1952	CA, CD, D, FW, L, P, PS, RE, RF, RI, ST, SW	C, E, NE, S, W	OL	18, 27, 30, 31, 33, 36, 38, 46, 49, 51, 84, 92, 109, 111, 112, 114
9	*E. phuphanensis* Sanoamuang, 2001	CA, P, RE, RF, RI, ST, SW	NE, W	OL	16, 18, 22, 23, 24, 25, 28, 29, 30, 33, 38, 46, 49, 71, 82, 84, 109, 111, 114, 115
10	*E. phuvongi* Sanoamuang & Sivongxay, 2004	CA, P, RF, RI	NE	OL	29, 30, 38, 71, 84, 86, 109
11	*E. sanoamuangae* Ranga Reddy & Dumont, 1998	CA, L, P, PL, RE, RF, RI, SW	C, N, NE, W	OL	11, 12, 14, 18, 21, 22, 24, 25, 35, 41, 75, 84, 99, 109, 111, 114, 115
12	*Heliodiaptomus*elegans Kiefer, 1935	CA, D, FW, L, P, RE, RF, RI, SW	C, N, NE, W	OL	12, 13, 14, 16, 18, 27, 30, 31, 33, 35, 37, 38, 46, 49, 81, 82, 83, 84, 109, 111, 114
13	*H. phuthaiorum* Sanoamuang, 2004	CA, P, RI	NE	OL	28, 84, 109, 115
14	*H. viduus* (Gurney, 1916)	CA, L, P, RE	C, N, NE, W	OL	3, 12, 14, 34, 37, 46, 49, 77, 84
15	*Mongolodiaptomus botulifer* (Kiefer, 1974) Remark: Previously recorded as *Neodiaptomus botulifer* (Boonsom, 1984).	CA, CD, CP, CS, D, FW, L, P, PL, PS, RE, RF, RI, ST, SW	C, E, N, NE, S, W	OL	2, 3, 4, 7, 8, 9, 12, 14, 17, 18, 19, 20, 21, 22, 23, 25, 26, 27, 29, 30, 31, 33, 35, 36, 37, 38, 40, 41, 46, 48, 49, 50, 51, 54, 57, 59, 63, 66, 71, 74, 75, 77, 81, 82, 83, 84, 92, 96, 97, 99, 103, 109, 111, 112, 114, 115
16	*M. calcarus* (Shen & Tai, 1965) Remark: Previously recorded as *Allodiaptomus calcarus* (Sanoamuang, 1994, 1999, 2001c), *Mongolodiaptomus mephistopheles* (Bricker et al., 1978; Lai and Fernando, 1981) and *Neodiaptomus mephistopheles* (Boonsom, 1984; Sanoamuang, 1994).	CA, FW, L, P, PS, RE, RF, RI, SW	C, N, NE, S, W	OL	2, 3, 4, 7, 12, 14, 17, 18, 21, 22, 24, 25, 27, 28, 30, 31, 33, 35, 38, 46, 49, 51, 74, 75, 77, 84, 99, 109, 114, 115
17	*M. dumonti* Sanoamuang, 2001	CA, CP, L, P, RE, RF, RI, ST, SW	C, E, N, NE, W	OL	17, 18, 19, 21, 23, 24, 30, 35, 36, 38, 46, 49, 75, 82, 84, 109, 111, 114
18	*M. loeiensis* Watiroyram & Sanoamuang, 2017*	P, RI	NE	OL	67, 74, 84, 109
19	*M. malaindosinensis* (Lai & Fernando, 1978)	CA, CD, CP, D, FW, L, P, PS, RE, RF, RI, ST, SW	C, E, N, NE, S, W	OL	3, 9, 17, 18, 19, 21, 23, 24, 25, 27, 28, 30, 31, 33, 38, 46, 48, 49, 50, 54, 71, 75, 77, 81, 82, 84, 86, 92, 109, 111, 114
20	*M. mekongensis* Sanoamuang & Watiroyram, 2018	CA, P, RF, RI	NE	OL, PA	71, 84, 109
21	*M. nakhonphanomensis* Watiroyram, Monongdern & Koompoot, 2025*	P	NE	OL	111
22	*M. pectinidactylus* (Shen & Tai, 1964)	CA, L, P, RF, RI	NE	OL, PA	24, 25, 30, 37, 38, 82, 83, 84, 109, 111, 114
23	*M. phutakaensis* Sanoamuang & Koompoot, 2024*	SW	NE	OL	105
24	*M. rarus* (Ranga Reddy, Sanoamuang & Dumont, 1998)* Remark: Previously recorded as *Allodiaptomus rarus* (Ranga Reddy et al., 1998; Sanoamuang, 1999).	CA, P, PL, RE, RF, RI, SW	NE	OL	12, 14, 17, 21, 22, 25, 28, 33, 84, 105, 109, 114, 115
25	*M. uenoi* (Kikuchi, 1936)	CA, FW, P, RE, RF, RI, SW	NE	OL, PA	12, 14, 15, 22, 23, 24, 25, 27, 30, 31, 33, 38, 71, 84, 114
26	*Neodiaptomus laii* Kiefer, 1974	CA, P, RF, RI, SW	E, NE, S	OL	3, 9, 19, 22, 23, 24, 25, 30, 36, 38, 71, 82, 84, 86, 109, 114
27	*N. meggitti* Kiefer, 1932	P	N	OL	75, 84
28	*N. schmackeri* (Poppe & Richard, 1892)	CA, P, PL, RE, RF, SW	C, N, NE, W	OL	3, 4, 12, 14, 35, 41, 46, 49, 77, 84, 99
29	*N. siamensis* Proongkiat & Sanoamuang, 2008*	PL	C	OL	41, 84
30	*N. songkhramensis* Sanoamuang & Athibai, 2002*	CA, P, PS, RF, SW	NE	OL	21, 22, 25, 28, 30, 33, 38, 82, 84, 86, 115
31	*N. yangtsekiangensis* Mashiko, 1951	CA, FW, L, P, PS, RE, RF, RI, ST, SW	E, NE, S	OL, PA	7, 12, 14, 18, 19, 22, 23, 24, 25, 27, 28, 30, 31, 33, 37, 38, 40, 59, 63, 71, 82, 84, 92, 109, 111, 114, 115
32	*Paradiaptomus greeni* (Gurney, 1906)	P	NE	AT, OL, PA	84
33	*Phyllodiaptomus christineae* Dumont, Ranga Reddy & Sanoamuang, 1996	CA, FW, L, P, RE, RF, RI, ST, SW	C, E, N, NE, W	OL	8, 12, 14, 18, 19, 23, 24, 27, 30, 31, 33, 34, 35, 38, 46, 49, 71, 77, 82, 84, 92, 96, 103, 114
34	*P. parachristineae* Sanoamuang & Watiroyram, 2023	P, RI	NE	OL	98, 109, 111
35	*P. praedictus* Dumont & Ranga Reddy, 1994 Remark: Previously misidentified as *Phyllodiaptomus annae* (Apstein, 1907) in earlier studies (Bricker et al. 1978; Lai and Fernando 1981; Boonsom 1984).	CA, CD, CP, CS, D, L, P, PL, RE, RF, RI, ST, SW	C, E, N, NE, S, W	OL	2, 3, 4, 6, 7, 12, 14, 17, 18, 21, 22, 24, 25, 28, 33, 35, 36, 37, 41, 42, 46, 49, 50, 54, 57, 74, 75, 77, 84, 92, 96, 99, 103, 105, 109, 111, 114, 115
36	*P. roietensis* Sanoamuang & Watiroyram, 2020	CA, P, PL, PS, RF	E, NE	OL	78, 82, 84, 114
37	*P. surinensis* Sanoamuang & Yindee, 2001*	CA, RF, SW	NE	OL	19, 23, 82, 84, 114
38	*P. thailandicus* Sanoamuang & Teeramaethee, 2006*	CA, P, PS, RI	C, E, NE, S, W	OL	36, 46, 49, 66, 74, 84, 109
39	*Tropodiaptomus doriai* (Richard, 1894) Remark: Previously recorded as *Diaptomus doriai* (Daday, 1906).	PL	C	OL	1
40	*T. hebereri* (Kiefer, 1930)	CA	N	OL	84
41	*T. lanaonus* Kiefer, 1982	L, RI, SW	C, NE	OL	33, 37, 84, 109
42	*T. lannaensis* Koompoot & Sanoamuang, 2025*	P	N	OL	108
43	*T. longiprocessus* Saetang & Maiphae, 2023*	SW	W	OL	97, 114
44	*T. megahyaline* Saetang, Sanoamuang & Maiphae, 2021*	L, P, RF, RI	NE	OL	82, 83, 84, 109, 114
45	*T. oryzanus* Kiefer, 1937	CA, FW, L, P, RF, RI, SW	NE	OL, PA	21, 22, 23, 24, 25, 27, 29, 30, 31, 33, 37, 38, 82, 84, 93, 109, 114
46	*T. pedecrassum* Saetang & Maiphae, 2023*	SW	N	OL	97, 114
47	*T. ruttneri* (Brehm, 1923)	PS, RF, RI, SW	N, NE, S	OL, PA	75, 82, 84, 109
48	*T. sirindhornae* Saetang & Maiphae, 2025*	CA, RF, RI, SW	E, NE, S	OL	110, 114
49	*T. vicinus* (Kiefer, 1930)	CA, FW, L, P, PS, RF, RI, ST, SW	C, E, N, NE, S, W	OL	3, 7, 14, 19, 23, 27, 31, 33, 46, 49, 81, 82, 84, 93, 109, 114
50	*Vietodiaptomus blachei* (Brehm, 1951) Remark: Previously recorded as *Neodiaptomus malaysianus* (Boonsom, 1984) and *N. blachei* (Sanoamuang, 1999); later recorded as *Vietodiaptomus blachei* (Sanoamuang & Watiroyram, 2018).	CA, CD, CP, D, FW, L, P, PL, RE, RF, RI, SW	C, N, NE, S, W	OL	2, 3, 4, 9, 14, 18, 20, 21, 22, 23, 24, 25, 27, 28, 29, 30, 31, 33, 34, 35, 38, 41, 46, 49, 71, 74, 75, 77, 82, 83, 84, 86, 99, 109, 111, 114, 115
	**Family Pseudocyclopidae Giesbrecht, 1892-1893**				
51	*Boholina laorsriae* Boonyanusith, Wongkamhaeng & Athibai, 2020*	CP	S	OL	76, 79
	**Family Pseudodiaptomidae Sars G.O., 1902**				
52	*Pseudodiaptomus dauglishi* Sewell, 1932	CA, RE	C, E, S	OL	3, 91, 114
	**Order Cyclopoida Burmeister, 1834**				
	**Family Cyclopettidae Martínez Arbizu, 2000**				
53	Limnoithona cf. sinensis	CA	C, S	-	32
	**Family Cyclopidae Rafinesque, 1815**				
54	*Acanthocyclops vernalis* (Fischer, 1853)	RE	-	NT, OL, PA	4
55	*Afrocyclops henrii* Alekseev & Sanoamuang, 2006*	CP, ST	C	OL	32, 52
56	*Apocyclops borneoensis* Lindberg, 1955	PL	E	OL, PA	95
57	*Bryocyclops asetus* Watiroyram, 2018*	CP	W	OL	72
58	*B. jayabhumi* Watiroyram, 2021*	CP	NE	OL	87
59	*B. maewaensis* Watiroyram, Brancelj & Sanoamuang, 2012*	CP	C, N, W	OL	52, 53, 54, 77, 99
60	*B. maholarnensis* Watiroyram, Brancelj & Sanoamuang, 2015*	CP	NE	OL	61, 74
61	*B. muscicola* (Menzel, 1926)	CP, GW, P	E, N, NE, S	NA, OL	72, 74, 75, 88, 102
62	*B. muscicoloides* Watiroyram, 2018*	CP	S	OL	73
63	*B. trangensis* Watiroyram, 2018*	CP	S	OL	73
64	*Cryptocyclops bicolor* (Sars G.O., 1863) Remark: Previously recorded as *Microcyclops bicolor* (Daday, 1906; Boonsom, 1984).	CA, CD, MI, PL, PS, RE, RI, ST, SW	C, N, NE, W, S	Cosmopolitan	1, 2, 4, 7, 14, 23, 33, 48, 109
65	*C. linjanticus* (Kiefer, 1928)	CA, FW, L, P, PS, SW	NE, S	AT, OL, PA	27, 31, 32, 48, 51
66	*Diacyclops nanus* (Sars G.O., 1863) Remark: Previously recorded as *Cyclops nanus* (Boonsom, 1984).	RE	-	OL, PA	4
67	*Ectocyclops medius* Kiefer, 1930	P	C	AT, OL	43
68	*E. phaleratus* (Koch, 1838) Remark: Previously recorded as *Cyclops paleratus* (Daday, 1906).	CA, P, PL, PS, RE, SW	C, E, N, NE, S	Cosmopolitan	1, 14, 35, 48, 114
69	*E. polyspinosus* Harada, 1931	CA, L, P, PS, RE, RI, ST	N, NE, S	OL, PA	32, 35, 37, 66, 109
70	*E. rubescens* Brady, 1904	CA, L, P, PS, RF, RI, SW	E, N, NE, W, S	Cosmopolitan	9, 14, 50, 51, 59, 63, 81, 114
71	Ectocyclops cf. mozhae	CP	C	-	52
72	*Eucyclops arcanus* Alekseev, 1990	L	NE	OL, PA	32
73	*E. euacanthus* (Sars G.O., 1909) Remark: *E. euacanthus cognatus* Kiefer, 1929	CA, P, RI	C, N, NE, S	OL, PA	32, 35, 77, 114
74	*E. microdenticulatus* Lindberg, 1940	P, PL, RE, ST	NE	Cosmopolitan	32
75	*E. pacificus* Ishida, 2000	P, ST	NE	OL, PA	32
76	*E. serrulatus* (Fischer, 1851) Remark: Historically regarded as cosmopolitan and reported from Thailand as *E. serrulatus*; however, *E. serrulatus* sensu stricto is currently considered restricted to the Palaearctic Region and Thai material has been referred to asE. cf. serrulatus. The identity of Thai records therefore needs further taxonomic verification (Alekseev and Sanoamuang 2006; Alekseev and Defaye 2011).	CA, CD, CP, FW, L, MI, P, PS, RE, RF, SW	C, E, N, NE, S, W	Cosmopolitan	7, 14, 23, 31, 37, 46, 48, 52, 54, 75, 77, 96, 114
77	Eucyclops cf. spatulatus	P	C	-	43
78	*Fierscyclops solaris* Boonyanusith, Brancelj & Sanoamuang, 2013*	CP, CS	S, W	OL	54, 55, 57
79	*F. tanaosriensis* Boonyanusith, Brancelj & Sanoamuang, 2013*	CP, CS	W	OL	54, 55
80	*Macrocyclops albidus* (Jurine, 1820)	ST	NE	Cosmopolitan	32
81	*M. distinctus* (Richard, 1887)	PS	S	AU, OL, PA	48
82	*M. fuscus* (Jurine, 1820)	L, ST	NE	Cosmopolitan	32
83	*M. neuter* Kiefer, 1931	ST, SW	NE	Cosmopolitan	32, 114
84	*Mesocyclops affinis* van de Velde, 1987	CA, CP, L, P, PS, RF, RI, SW	C, E, N, NE, S, W	AU, OL	46, 50, 51, 52, 54, 59, 63, 65, 66, 75, 77, 96, 99, 103, 104, 109, 111, 114
85	*M. aequatorialis* Kiefer, 1929	CD, MI, RE, PS, SW	S	AU, OL, PA	48
86	*M. aspericornis* (Daday, 1906)	CA, CD, CP, CL, FW, L, P, PS, RE, RF, RI, SW	C, E, N, NE, S, W	AT, AU, NT, OL	7, 14, 21, 23, 24, 25, 27, 30, 31, 32, 33, 35, 37, 38, 46, 50, 51, 57, 59, 63, 75, 77, 96, 99, 103, 104, 109, 114
87	*M. dadayi* Holynska, 1997	ST	S	OL	47
88	*M. dissimilis* Defaye & Kawabata, 1993	L	-	OL, PA	35
89	*M. ferjemurami* Holynska & Nam, 2000	CA, P, RF, RI, SW	C, N, NE	OL	30, 35, 38, 109, 114
90	*M. francisci* Holynska, 2000	CP, PS	C, E, S	OL	51, 52, 114
91	*M. kayi* Holynska & Brown, 2003	RF, RI	C, NE	OL	96, 109
92	*M. leuckarti* (Claus, 1857) Remark: Previously recorded as *Cyclops leuckarti* (Daday, 1906).	CA, CD, GW, PL, RE, SW	C, NE, S, W	AU, OL, PA	1, 2, 5, 48
93	*M. microlasius* Kiefer, 1981	CD, CP, PS, SW	C, N, S	AU, OL, PA	48, 51, 52, 114
94	*M. mongoliensis* Kiefer, 1981	RF	N	OL, PA	75
95	*M. ogunnus* Onabamiro, 1957	CA, CP, GW, L, P, PL, PS, RF, RI, ST, SW	C, E, N, NE, S, W	AT, NT, OL, PA	32, 46, 47, 50, 51, 54, 59, 63, 66, 75, 77, 95, 96, 99, 103, 104, 109, 114
96	*M. papuensis* van de Velde, 1987	CP, L, PS, SW	E, N, NE, S	AU, OL	50, 57, 59, 63, 66, 114
97	*M. pehpeiensis* Hu, 1943	L, P, ST, SW	N, NE, W	NT, OL, PA	32, 35, 37, 114
98	*M. pilosus* Kiefer, 1930	CA	N	AT, OL	35
99	*M. shenzhenensis* Guo, 2000	CP	S	OL, PA	57
100	*M. splendidus* Lindberg, 1943	SW	NE	OL	14, 114
101	*M. thermocyclopoides* Harada, 1931	CA, CD, CP, CS, D, FW, L, MI, P, PS, RE, RF, RI, ST, SW	C, E, N, NE, S, W	NT, OL, PA	7, 9, 10, 14, 20, 21, 23, 24, 25, 26, 27, 30, 31, 32, 33, 34, 35, 37, 38, 42, 46, 47, 48, 50, 51, 54, 59, 63, 66, 67, 75, 77, 81, 92, 96, 99, 103, 104, 109, 111, 112, 114
102	*M. woutersi* van de Velde, 1987	P, PS, RI, SW	N, NE, S	AU, OL, PA	109, 114
103	Mesocyclops cf. parentium	CP, CS	S	OL, PA	57
104	*Metacyclops brancelji* Athibai, Wongkamhaeng & Boonyanusith, 2022*	CP	S	OL	90
105	*M. sakaeratensis* Athibai, Wongkamhaeng & Boonyanusith, 2022*	ST	NE	OL	90
106	*Microcyclops karvei* Kiefer & Moorthy, 1935	CA, PS, RI	C, NE, S	OL, PA	66, 77, 109
107	*M. pachyspina* Lindberg, 1937	P, PS, RI	NE, S, W	OL, PA	46, 51, 109
108	*M. rubellus* (Lilljeborg, 1901)	CP, P, RE, RI	N, NE	Cosmopolitan	32, 75, 109, 111
109	*M. varicans* (Sars G.O., 1863)	CA, CD, CP, FW, GW, L, MI, P, PS, RE, RF, RI, ST, SW	C, E, N, NE, S, W	Cosmopolitan	2, 4, 7, 9, 14, 23, 27, 31, 32, 33, 46, 48, 54, 57, 66, 75, 77, 81, 92, 99, 114
110	*Paracyclops affinis* (Sars G.O., 1863)	CA, FW, L, P, PS, RE, RF, RI, SW	C, E, NE, S	Cosmopolitan	27, 31, 32, 48, 63, 96, 109, 114
111	*P. fimbriatus* (Fischer, 1853) Remark: Previously recorded as *Cyclops fimbriatus* (Daday, 1906) and *Paracyclops vagus* (Alekseev and Sanoamuang, 2006).	CA, CP, L, P, PL, RE, RF, RI, ST, SW	C, N, NE, S, W	Cosmopolitan	1, 14, 23, 32, 35, 50, 52, 54, 57, 59, 63, 75, 77, 114
112	*Pseudohesperocyclops loeiensis* Brancelj, Boonyanusith & Sanoamuang, 2024*	GW	NE	OL	102
113	*P. thailandicus* (Boonyanusith, Sanoamuang & Brancelj, 2018)*	CP	W	OL	69
114	*Siamcyclops cavernicolus* Boonyanusith, Sanoamuang & Brancelj, 2018*	CP	C, W	OL	69, 77
115	*S. isanus* Watiroyram & Koompoot, 2024*	CP	NE	OL	106
116	*Thermocyclops crassus* (Fischer, 1853) Remark: Previously recorded as *Mesocyclops crassus* (Bricker et al., 1978)	CA, CD, CP, CS, FW, L, P, PS, RE, RF, RI, ST, SW	C, E, N, NE, S, W	Cosmopolitan	2, 7, 9, 14, 21, 24, 25, 26, 27, 30, 31, 33, 34, 35, 37, 38, 46, 48, 50, 51, 54, 57, 59, 63, 66, 75, 77, 79, 92, 96, 103, 104, 109, 111, 114
117	*T. decipiens* (Kiefer, 1929)	CA, CP, D, FW, L, MI, P, PS, RE, RF, RI, ST, SW	C, N, NE, S, W	Cosmopolitan	7, 14, 23, 25, 27, 30, 31, 32, 33, 35, 37, 38, 42, 46, 48, 50, 51, 54, 57, 66, 67, 75, 77, 81, 96, 99, 103, 104, 109, 111, 114
118	*T. incisus* (Kiefer, 1932)	CA, P, RE	N, W	AT, OL	35
119	*T. maheensis* Lindberg, 1941	RI, SW	N, NE	OL	32, 109, 114
120	*T. oblongatus* (Sars G.O., 1927)	P, RE	C, N	AT, OL, PA	35
121	*T. oithonoides* (Sars G.O., 1863) Remark: Previously recorded as *Cyclops oithonoides* (Daday, 1906).	PL	C	OL, PA	1
122	*T. operculifer* Kiefer, 1930	CA, CP, P, RF, RI	C, N, S, W	OL	52, 54, 57, 77, 96, 99, 104
123	*T. oryzae* Saetang, Koompoot, Watiroyram & Maiphae, 2024*	CA, P, RF, RI, SW	C, NE	OL	103, 104, 109, 114
124	*T. parahastatus* Karanovic, Koomput & Sanoamuang, 2017*	CP	N	OL	65, 75, 80
125	*T. rylovi* (Smirnov, 1928)	CA, P, RF, RI, SW	C, N, NE, W	OL	32, 46, 77, 96, 103, 104, 109, 114
126	*T. schmeili* (Poppe & Mrázek, 1895)	CP	S	AT, OL	57
127	*T. taihokuensis* Harada, 1931	CA, D, L, P, RE, RF, RI, ST	C, N, NE, W	OL, PA	7, 14, 35, 46, 75, 96
128	*T. thailandensis* Karanovic, Koomput & Sanoamuang, 2017*	CP	N	OL	65, 75, 80
129	*T. vermifer* Lindberg, 1935	CA, CP, P, RE, RF, RI, SW	C, NE, W	OL, PA	46, 77, 96, 99, 104, 109
130	*T. wolterecki* Kiefer, 1938	CA, L, P, PS, RF, RI	C, E, N, NE, S	AU, OL	32, 35, 77, 96, 103, 104, 109, 111, 114
131	Thermocyclops cf. tinctus	CL, CS	S	AT, OL	57
132	*Tropocyclops confinis* (Kiefer, 1930)	P, PL, RE, RI	N, NE	AU, NA, OL	32, 35
133	*T. laorsriae* Koompoot & Phlai-Ngam, 2026*	P	E	OL	113
134	*T. prasinus* (Fischer, 1860)	CA, CD, CL, CP, CS, L, MI, P, PL, PS, RE, RF, RI, ST, SW	C, E, N, NE, S	Cosmopolitan	2, 4, 14, 23, 32, 35, 47, 48, 52, 57, 75, 114
135	*T. tenellus* (Sars G.O., 1909)	GW	NE	AU, NA, OL	32
	**Family Halicyclopidae Kiefer, 1927**				
136	Halicyclops cf. thermophilus	CA	C, S	-	32, 35
137	*Neocyclops* sp.	-	-	-	32
	**Order Harpacticoida Sars G.O., 1903**				
	**Family Ameiridae Boeck, 1865**				
138	*Nitocra lacustris* (Schmankevitsch, 1875)	CP	NE	NA, NT, OL, PA	74
139	*Nitocrella grandicaudis* Boonyanusith, Brancelj & Sanoamuang, 2025*	CS	NE	OL	107
140	*N. thailandensis* Boonyanusith, Brancelj & Sanoamuang, 2025*	CS	NE	OL	107
141	*Nitocrellopsis* sp.	CP	S	-	57
142	*Parapseudoleptomesochra phayaoensis* Koompoot & Sanoamuang, 2021*	CP	N	OL	80
	**Family Canthocamptidae Brady, 1880**				
143	*Attheyella thailandica* Watiroyram, 2021*	CP	E, NE, S	OL	88, 99, 101
144	*A. vietnamica* Borutzky, 1967	CP	C, NE	OL	52, 62, 74, 99
145	Bryocamptus cf. echinatus	CP	C, N	OL	52, 62
146	*Cletocamptus thailandensis* Boonyanusith & Wongkamhaeng, 2023*	PL	E	OL	95
147	*Elaphoidella bidens* (Schmeil, 1893) Remark: Recorded in Thailand as *Elaphoidella bidens decorata* (Daday, 1901).	CD, CL, CP, CS, P, PS, ST, SW	E, N, NE, S	Cosmopolitan	56, 57, 74, 75, 99, 114
148	*E. brancelji* Sanoamuang & Watiroyram, 2021*	CP	C	OL	85
149	*E. bromeliaecola* (Chappuis, 1928)	CP, CS, P	C, N, NE, S, W	AT, OL	52, 54, 57, 62, 74, 75
150	*E. grandidieri* (Guerne & Richard, 1893) Remark: Previously recorded as *Attheyella grandidieri* (Daday, 1906).	PL	C	AT, OL, PA, NT	1
151	*E. intermedia* Chappuis, 1931	CA, CP, SW	C, N, NE, W	AT, OL	52, 62, 74, 75, 77, 114
152	*E. isana* Watiroyram, Sanoamuang & Brancelj, 2021*	CP, GW	C, NE	OL	77, 89, 101, 102
153	*E. jaesornensis* Watiroyram, Brancelj & Sanoamuang, 2015*	CP	N	OL	62
154	*E. ligorae* Watiroyram, Sanoamuang & Brancelj, 2017*	CP	S	OL	68
155	*E. longiramus* Watiroyram, Janpong & Sanoamuang, 2022*	CP	C	OL	94
156	*E. margaritae* Pesce & Apostolov, 1985*	GW	S	OL	5
157	*E. namnaoensis* Brancelj, Watiroyram & Sanoamuang, 2010*	CL, CP, CS, P, ST	C, N, NE, S, W	OL	44, 52, 54, 56, 57, 74, 75, 77
158	*E. paraaffinis* Watiroyram, Sanoamuang & Brancelj, 2017*	CP	S	OL	68
159	*E. phuphamanensis* Boonyanusith, Brancelj & Sanoamuang, 2024*	CP	NE	OL	101
160	*E. propecabezasi* Boonyanusith, Brancelj & Sanoamuang, 2024*	CS	NE	OL	101, 107
161	*E. sanoamuangae* Watiroyram & Brancelj, 2016 *	CP	S	OL	64
162	*E. stygobiotica* Watiroyram & Koompoot, 2023*	CP	W	OL	100
163	*E. superpedalis* Shen & Tai, 1964	CA	NE	OL, PA	114
164	*E. thailandensis* Watiroyram, Brancelj & Sanoamuang, 2015*	CP	C	OL	62
165	Elaphoidella cf. sewelli	CU	S	-	57
166	*Epactophanes richardi* Mrázek, 1893	CL, CP, CS	C, N, NE, S, W	AT, OL	52, 54, 57, 74, 77
167	*Maraenobiotus* sp.	CP	NE	-	74
168	*Mesochra* sp.	L	S	-	63
169	*Moraria* sp.	CP	C	-	52
170	*Speleocamptus* sp.	CP	W	-	54
	**Family Ectinosomatidae Sars G.O., 1903**				
171	*Halectinosoma* sp.	L	S	-	63
172	*Rangabradya wongkamhaengae* Boonyanusith & Athibai, 2021*	CP	S	OL	79
	**Family Laophontidae Scott T., 1905**				
173	*Onychocamptus bengalensis* (Sewell, 1934)	CU, PS	E, S	AU, OL	70
174	*O. mohammed* (Blanchard & Richard, 1891) Remark: Previously recorded as *Laophonte mohammed* (Daday, 1906).	CP, PL, PS	C, E, S	Cosmopolitan	1, 39, 52, 66, 70, 114
175	*O. satunensis* Boonyanusith, Saetang, Wongkamheng & Maiphae, 2018*	CL	S	OL	70
176	*O. tratensis* Boonyanusith, Saetang, Wongkamheng & Maiphae, 2018*	PS	E	OL	66, 70, 114
177	*O. vitiospinulosa* (Shen & Tai, 1963)	L, RF	C, S	OL, PA	63, 70, 96
178	Onychocamptus cf. anomalus	CS	S	-	57
	**Family Miraciidae Dana, 1846**				
179	*Schizopera paktaii* Watiroyram, Sanoamuang & Brancelj, 2021*	CP	S	OL	89
	**Family Parastenocarididae Chappuis, 1940**				
180	*Asiacaris dispar* Cottarelli, Bruno & Berera, 2010*	GW	S	OL	45
181	*Kinnecaris iulianae* Bruno & Cottarelli, 2015*	GW	S	OL	58
182	*Parastenocaris* sp.	PS	S	-	66
	**Family Phyllognathopodidae Gurney, 1932**				
183	*Neophyllognathopus* sp.	CU	S	-	57
184	*Phyllognathopus inexspectatus* Galassi & De Laurentiis, 2011	CS	S	OL, PA	57
185	*P. paludosus* Mrázek, 1893	L, PL, PS, RF	E, S	AT, OL, PA	59, 63, 95, 114
186	*P. viguieri* (Maupas, 1892)	CP, CS, PS, ST	NE, S, W	Cosmopolitan	54, 56, 57, 66, 74
	**Family Tegastidae Sars G.O., 1904**				
187	*Parategastes pholpunthini* Saetang & Maiphae, 2015*	CA	S	OL	60, 63
